# Perceived stress and its associated factors among people living in post-war Districts of Northern Ethiopia: A cross-sectional study

**DOI:** 10.1371/journal.pone.0279571

**Published:** 2022-12-28

**Authors:** Mesfin Tadese, Saba Desta Tessema, Abebe Mihretie, Getu Engida Wake, Hana Nigussie Teshome, Getaneh Baye Mulu, Tesfa Dejenie Habtewold

**Affiliations:** 1 Department of Midwifery, School of Nursing and Midwifery, Asrat Woldeyes Health Science Campus, Debre Berhan University, Debre Berhan, Ethiopia; 2 Department of Nursing, School of Nursing and Midwifery, Asrat Woldeyes Health Science Campus, Debre Berhan University, Debre Berhan, Ethiopia; 3 Branch of Epidemiology, Division of Intramural Population Health Research, *Eunice Kennedy Shriver* National Institute of Child Health and Human Development, National Institutes of Health, Bethesda, Maryland, United States of America; 4 Department of Epidemiology, University Medical Centre Groningen, University of Groningen, Groningen, The Netherlands; University of Concepcion Faculty of Medicine: Universidad de Concepcion Facultad de Medicina, CHILE

## Abstract

**Background:**

War and conflict environments result in long-term physical and psychological consequences. Sexual violence, displacement, malnutrition, death, illness, injury, torture, and disability are some of the physical effects, whereas stress, depression, aggressive behaviors, and anxiety are some of the emotional complications of war. Hence, evidence-based interventions are required particularly to monitor mental health disorders. Thus, we aimed to investigate the prevalence of perceived stress and its associated factors among people living in post-war situations, Northern Ethiopia.

**Method:**

A community-based cross-sectional study design was employed among 812 samples from April 1 to May 15, 2022. The study participants were selected using a multistage sampling technique. The data was collected through face-to-face interviews using a structured and pre-tested tool. Data were cleaned and entered into Epi-Data version 4.6 and transferred to SPSS version 25 for analysis. Binary logistic regression analysis was performed to identify determinants of perceived stress. The Hosmer-Lemeshow goodness-of-fit was applied to test for model fitness and a p-value of <0.05 was considered statistically significant.

**Result:**

The prevalence of perceived stress was 76.1%, 95% CI (72.9–78.8). Age above 45 years (AOR (CI) = 2.45 (1.07–5.62), poor educational level (AOR (CI) = 5.92 (2.36–14.8), large family size (AOR (CI) = 0.48 (0.31–0.74), alcohol consumption (AOR (CI) = 0.63 (0.42–0.94), smoking (AOR (CI) = 0.17 (0.06–0.56), and exposure to multiple traumatic events (AOR (CI) = 2.38 (1.23–4.62) have shown a statistically significant association with perceived stress.

**Conclusion:**

This study revealed that more than three-fourths of participants living in post-war settings were found to have perceived stress. Older age, poor level of education, large family size, alcohol consumption, smoking, and the number of traumatic events were significant associates of perceived stress. Psychotherapy that can effectively address the medical, social, and psychological well-being of the community is important to reduce the burden of perceived stress.

## Background

There is a conflict between the Ethiopian federal government and Tigray rebels, called the Tigray Peoples Liberation Front (TPLF), in the Northern part of Ethiopia. The war started on Nov 4, 2020, as the TPLF attacked the federal government military bases (South division) located in the Tigray region. TPLF is listed as a terrorist organization by Ethiopia and several states and organizations. During this period, TPLF was responsible for the vast majority of terrorism-related events in these regions. The violence and conflict environment has resulted in massive displacement, homelessness, financial and family loss, thousands of loss of life, and disruption of the culture and values of millions of people [1].

People exposed to various traumatic life events during the war can experience both physical and psychological consequences directly or indirectly. Sexual violence, malnutrition, death, illness, injury, torture, and disability are some of the physical effects, whereas stress, depression, aggressive behaviors, and anxiety are some of the emotional complications of war [2]. In the two most affected regions, Afar and Amhara, more than 28,560 individuals developed severe forms of mental health problems that require immediate intervention, of whom 14,565 are women and 12,566 are children. Treatment of these victims has been difficult, as more than 1500 health facilities have been destroyed and about 10,000 health care workers have been displaced. These events aggravate the mental health disorders of the communities living in the war-affected areas and indirectly affect their future generations [1].

Stress is a stimulus that produces mental tension or physiological reaction in response to a specific traumatic event or stressor [3]. Stress remains the most prevalent psychological outcome following the war and political violence. The prevalence of perceived stress in Nepal was 65.1% [4], while 30% in Saudi Arabia [5] and 76.76% in west Africa [6]. In Jimma and Southern Ethiopia, the level of perceived stress was determined at 35.9% [7] and 61.8% [8], respectively.

Although the exact cause of most mental illnesses is mostly unknown, the combination of biological, psychological, and environmental factors is thought to play a substantial role [9]. Not getting along well with people were positively associated with perceived stress, while, feeling overwhelmed by the demand of everyday life and worrying about what other people think about them were negatively associated with perceived stress [8]. Decreased household income and religion as means of coping with stress increased the risk of perceived stress [7]. In addition, perceived stress was significantly higher among females [10] and those with chronic illnesses [5]. Younger age and attending a higher level of education were also recognized as risk factors for developing stress [11].

Stress imposes a significant burden on the public. Increased stress levels are related to decreased job performance, higher medical costs, and poor relationships including family breakdowns leaving children unattended [12]. It also disrupts the whole life, health, and enjoyment of everyday activities [1]. In addition, a high level of stress substantially increases the risk of multiple cardiovascular diseases i.e., hypertension, diabetes, and obesity [13]. This makes stress highly associated with a poor quality of life even after the end of the actual hostilities in a post-armed conflict setting [14].

There are positive/healthy and negative/unhealthy coping strategies with stress. Healthy coping mechanisms include seeking support from a counselor or friend, cultural and religious ceremonies, meditation, journaling, and exercising, and they contribute to long-lasting positive outcomes [15]. On the other hand, unhealthy ways of coping mechanisms include attacking others and making them uncomfortable, avoiding a person, place, or thing, and substance use, i.e., khat chewing, smoking, and alcoholism [16]. These often provide instant relief but have long-term negative consequences by themselves. To lead a mentally healthy lifestyle, coping mechanisms need to be positive. Some people adapt to or cope with all the stresses, and others find it difficult. The degree and number of trauma or stressor, availability of resources, the type of coping strategy, and availability of social support determine whether an individual can adapt to or recover from stress [15].

Despite its common occurrence, stress persists invisible and is given low priority in most parts of the world including Ethiopia compared to other health problems. Besides, research is scarce about perceived stress in post-armed conflict regions. So, attention needs to be given to better prevention and management practices and to improve the quality of life and productivity. Thus, there is a need to investigate the prevalence of perceived stress and its associated factors among people living in post-conflict situations.

## Method and materials

### Study setting, design, and period

A community-based cross-sectional study design was employed from April 1 to May 15, 2022, in the North Shewa Zone of the Amhara region. The Zone is bordered on the south and the west by the Oromia region, on the north by South Wollo, and on the east by the Afar region. Based on the 2007 Census conducted by the Central Statistical Agency (CSA), North Shewa Zone has a total population of 1,837,490, of whom 908,796 women. The zone has 24 districts/woredas. Of these, 10 districts (Menz Gera Midir, Menz Lalo Midir, Menz Mama Midir, Menz Keya Midir, Geshe Rabel, Efratana Gidim, Kewot, Antsokiyana Gemza, Mojana Wadera, and Tarmaber) were invaded by TPLF during the war.

### Population and eligibility criteria

All people living in war-affected districts of the North Shewa Zone were the source population. All people aged above 18 years irrespective of their sex and who lived for a minimum of six months in the selected districts were included. Individuals who have a hearing problem, severe mental illness or are seriously ill and those who migrated or were not available at the time of invasion/war were excluded from the study.

### Sample size determination and sampling procedure

The sample size was determined using Open-Epi version 3.03 statistical software. The following assumptions were made: the power of the study (1-β) to be 80%, 95% confidence interval (CI), 5% margin of error, the prevalence of perceived stress, 51.6% [11], and design effect 2. Then, adding a 10% non-response rate, the sample size equals 845.

The study participants were selected using a multistage sampling technique. Four districts were selected using simple random sampling (SRS) and the calculated sample size was distributed to each of the selected districts based on the number of households using the probability proportional to size (PPS) method. The number of households in the selected districts was obtained from the administrative office of the district. Then, interval sampling was done to identify the required households from the predetermined sampling frame of all households in each district. Three times visits to participants were done for absence in the first visit.

### Study variables

Perceived stress was a dependent variable. Independent/exposure variables include socio-demographic factors (age, educational level, marital status, occupation, living arrangement, and family size), pre-existing factors (chronic illness, exposure to childhood trauma, stressful life events, self and family history of mental illness, sleeping hours and substance use), and trauma-related factors (destruction of personal property, lack of food, water and or shelter, witnessing the murder of a family/friends, witnessing the murder of stranger, ill health without medical care, forced isolation from family/other people, tortured or beaten, made to accept ideas against the will, unnatural death of family, friends, or people you love, being abducted or kidnapped or imprisoned, and rape or sexual abuse), and social support (S1 and S2 Files).

### Measurement

#### Perceived stress level

Measured using the Perceived Stress Scale (PSS). The questions in this scale asked about feelings and thoughts in the last month. PSS was measured with a 5-point Likert- scale ranging from 0 = Never, 1 = Almost never, 2 = Sometimes, 3 = Fairly often, 4 = Very often) and individuals with higher scores indicated higher perceived stress [17]. A total score of greater than or equal to 8 points was deemed as the cut-off point for categorizing perceived stress associated with war.

#### Social support

Was measured according to the Oslo-3 social support scale which ranges from 3 to 14, those respondents who scored between 3 and 8 were considered to have poor social support, a score of 9–11 was considered as having moderate social support, and a score of 12–14 was considered as having strong social support [18].

### Data collection tool and procedure

The data were collected through face-to-face interviews using a structured and pre-tested questionnaire at the participant’s home. The tool was adapted from the WHO and other measurement tools used in previous studies [5, 7, 10, 11] (S1 File). The interview took an average of 20–25 minutes.

Five data collectors and two supervisors were involved in the data collection. The data collectors and supervisors were trained for two days about the objective, approach, data collection tool, and techniques of household selection by the principal investigators. Back and forth questionnaire translations (from English to Amharic and vice versa) were performed to check the consistency of the questions with the original meaning (S1 and S2 Files). Before the actual data collection, a pre-test was done on 5% of the estimated sample size (42 households) at Debre Sina kebele and the required amendments were considered following the result. The supervisors and principal investigators closely followed the completeness, coherence, and clarity of the data across the entire data collection period.

### Data processing and analysis

The data were cleaned, recoded, and entered into Epi-Data version 4.6 and transferred to IBM SPSS version 25.0 statistical software for analysis. The principal investigator randomly picked one questionnaire and compared it with the corresponding entered data for quality control. Descriptive findings were presented using narratives, figures, and tables. Binary logistic regression analysis was run to identify independent predictors of perceived stress. Variables with a p-value of ≤0.25 in the bivariable regression analysis were included in the final multivariable logistic regression analysis model. Hosmer and Lemeshow’s goodness-of-fit were applied to test for model fitness. The multicollinearity between explanatory variables was also checked using variance inflation factor and tolerance and found within a tolerable range. The strength of association was interpreted using an adjusted odds ratio with a two-sided 95% confidence interval and the statistical significance was declared at a p-value of <0.05.

### Ethical approval

Ethical approval was obtained from the Institutional Review Board of Debre Berhan University, Asrat Woldeyes Health Science Campus. A formal support letter was written to the study districts. The data collectors explained the objective, potential risks, and benefits of the study. The participation was voluntary and informed written consent was obtained from the study participants. Those who cannot read and write were asked to thumbprint the consent form after the information was read. Confidentiality and anonymity were also assured.

## Result

### Baseline characteristics

A total of 812 participants were interviewed, making a 96.1% response rate. The mean (±SD) age of respondents was 31.13 ± 9.62 and ranged from 18 to 75 years. More than half, 428 (52.7%) of the respondents were found in the age range of 25–34 years. Nearly half of the participants 420 (51.7%) were married and 626 (77.1%) of them have four or fewer family sizes. Regarding the occupation of participants, 338 (41.6%) of them are government employees followed by private employees 174 (21.4) (Table 1).

**Table 1:** Distribution of socio-demographic data among people living in war-affected districts of Northern Ethiopia, 2022.

Variables	Category	Number	Percentage (%)
**Age**	18–24 years	172	21.2
	25–34 years	428	52.7
	35–44 years	124	15.3
	≥ 45 years	88	10.8
**Sex**	Male	470	57.9
	Female	342	42.1
**Residence**	Rural	80	9.9
	Urban	372	90.1
**Religion**	Christian	590	72.7
	Muslim	222	27.3
**Marital status**	Married	420	51.7
	Single	304	37.4
	Widowed	38	4.7
	Divorced	50	6.2
**Educational status**	No formal education	104	12.8
	Primary education	142	17.5
	Secondary education	172	21.2
	Higher education	394	48.5
**Occupation**	Farmer	104	12.8
	Housewife	58	7.1
	Government employee	338	41.6
	Private employee	174	21.4
	Daily laborer	70	8.6
	Student	36	4.4
	Jobless	32	3.9
**Living arrangement**	Live alone	254	31.3
	Live with wife/ children	442	54.4
	Live with others	116	14.3
**Family size**	≤4	626	77.1
	>4	186	22.9

### Pre-existing chronic illness and behavioral characteristics

Forty-four (5.4%) of participants had a history of childhood physical and/or sexual abuse. In addition, more than three-forth (77.6%) of respondents experienced adequate sleep, 236 (29.1%) consume alcohol, and 22 (2.7%) had a medically diagnosed history of mental illness. Nearly one-third, 282 (34.7%) and 640 (78.8%) of participants had poor social support and experienced more than three traumatic events, respectively (Table 2).

**Table 2:** Pre-existing chronic illness and behavioral characteristics of participants living in war-affected districts, Northern Ethiopia, 2022.

Variables	Category	Number	Percentage (%)
Pre-existing chronic illness	Yes	116	14.3
	No	696	85.7
Types of chronic illness	Hypertension	18	2.2
	Diabetes	18	2.2
	Cardiac disease	12	1.5
	Kidney disease	28	3.4
	Respiratory disease	10	1.2
	HIV/AIDS	16	2.0
	Other	30	3.7
Childhood physical and sexual abuse and neglect	Yes	44	5.4
	No	768	94.6
Alcohol use	Yes	236	29.1
	No	576	70.9
Khat use	Yes	80	9.9
	No	732	90.1
Smoking	Yes	24	3.0
	No	788	97.0
Sleeping hours	Inadequate (<7 hours)	182	22.4
	Adequate (≥7 hours)	630	77.6
Medically diagnosed history of mental illness	Yes	22	2.7
	No	790	97.3
Types of mental illness experienced	Depression	4	0.5
	Anxiety	6	0.7
	Substance abuse	4	0.5
	Stress disorder	8	1.0
	Others	6	0.7
Family history of mental illness	Yes	20	2.5
	No	792	97.5
Friends/family who died from mental illness	Yes	42	5.2
	No	770	94.8
Fight with family, friends, or people you love in the past month	Yes	116	14.3
	No	696	85.7
Social support	Poor	282	34.7
	Moderate	296	36.5
	Strong	234	28.8
Number of traumatic events	No events	44	5.4
	1–2 events	128	15.8
	≥ 3 events	640	78.8

### Trauma-related characteristics

Nearly three-fourths (73.4%) of respondents’ personal property was destructed during the war. In addition, 75.9% of them faced a lack of food and/or water, 37.4% witnessed the murder of a family member/friend, 52.5% were unable to access medical care, and 31% of participants were made to accept ideas against their will (Table 3).

**Table 3:** Distribution of trauma-related characteristics of participants living in war-affected districts, Northern Ethiopia, 2022.

Traumatic events encountered	Yes, n (%)	No, n (%)
Destruction of personal property	596 (73.4)	216 (26.6)
Lack of housing or shelter	488 (60.1)	324 (39.9)
Lack of food and/or water	616 (75.9)	196 (24.1)
Witness murder of family member/friends	304 (37.4)	508 (62.6)
Witnessing the murder of a stranger	322 (39.7)	490 (60.3)
Ill health without medical care	426 (52.5)	386 (47.5)
Forced isolation from family/other people	184 (22.7)	628 (77.3)
Tortured or beaten	234 (28.8)	578 (71.2)
Made to accept ideas against the will	252 (31.0)	560 (69.0)
Unnatural death of family, friends, or people you love	498 (61.3)	314 (38.7)
Being abducted or kidnapped or imprisoned	240 (29.6)	572 (70.4)
Rape or sexual abuse	132 (16.3)	680 (83.7)

### Prevalence and associated factors of perceived stress

The prevalence of perceived stress was 618 (76.1%), 95% CI (72.9–78.8). In the multivariable logistic regression analysis model, age, educational level, family size, alcohol consumption, smoking, and exposure to traumatic events have shown a statistically significant association with perceived stress (Table 4).

**Table 4:** Bivariable and multivariable logistic regression analysis of variables with perceived stress, Northern Ethiopia, 2022.

Variables	Perceived Stress		COR (95% CI)	AOR (95% CI)
	No	Yes		
**Age**				
18–24 years	52 (26.8)	120 (19.4)	1	1
25–34 years	108 (55.7)	320 (51.8)	1.28 (0.87–1.90)	1.22 (0.80–1.86)
35–44 years	20 (10.3)	104 (16.8)	2.25 (1.26–4.02)	1.60 (0.86–3.00)
≥ 45 years	14 (7.2)	74 (12.0)	2.29 (1.18–4.42)	2.45 (1.07–5.62)*
**Educational level**				
No formal education	6 (3.1)	98 (15.9)	6.82 (2.91–15.9)	5.92 (2.36–14.8)*
Primary education	24 (12.4)	118 (19.1)	2.05 (1.26–3.35)	1.58 (0.90–2.78)
Secondary education	48 (24.7)	124 (20.1)	1.08 (0.72–1.60)	0.99 (0.65–1.54)
Higher education	116 (59.8)	278 (45.0)	1	1
**Family size**				
≤4	138 (71.1)	488 (79.0)	1	1
≥5	56 (28.9)	130 (21.0)	0.66 (0.46–0.95)	0.48 (0.31–0.74)*
**Pre-existing chronic illness**				
Yes	22 (11.3)	94 (15.2)	1.40 (0.86–2.30)	0.77 (0.44–1.38)
No	172 (88.7)	524 (84.8)	1	1
**Childhood physical and sexual abuse and neglect**				
Yes	6 (3.1)	38 (6.1)	2.05 (0.85–4.93)	1.67 (0.61–4.55)
No	188 (96.9)	580 (93.9)	1	1
**Alcohol consumption**				
Yes	64 (33.0)	172 (27.8)	0.78 (0.55–1.11)	0.63 (0.42–0.94)*
No	130 (67.0)	446 (72.2)	1	1
**Khat use**				
Yes	14 (7.2)	66 (10.7)	1.54 (0.84–2.80)	2.02 (0.90–4.52)
No	180 (92.8)	552 (89.3)	1	1
**Smoking**				
Yes	10 (5.2)	14 (2.3)	0.43 (0.19–0.98)	0.17 (0.06–0.56)*
No	184 (94.8)	604 (97.7)	1	1
**Number of traumatic events**				
No events	18 (9.3)	26 (4.2)	1	1
1–2 events	54 (27.8)	74 (12.0)	0.95 (0.47–1.90)	0.85 (0.41–1.77)
≥ 3 events	122 (62.9)	518 (83.8)	2.94 (1.56–5.53)	2.38 (1.23–4.62)*

*Statistically significant at p-value <0.05

Respondents aged 45 years and above were two times more likely to develop perceived stress compared to those aged between 18–24 years (AOR (CI) = 2.45 (1.07–5.62). There were six times more odds of perceived stress among uneducated participants (AOR (CI) = 5.92 (2.36–14.8). Perceived stress was also two times more common in respondents who encountered more than three traumatic events (AOR (CI) = 2.38 (1.23–4.62). The odds of perceived stress were 37% lower among alcohol consumers than their counterparts (AOR (CI) = 0.63 (0.42–0.94). Additionally, smoking decreased the risk of perceived stress by 83% (AOR (CI) = 0.17 (0.06–0.56). Further, there are 52% decreased odds of developing perceived stress among participants who had more than four family sizes (AOR (CI) = 0.48 (0.31–0.74) (Table 4).

## Discussion

The prevalence of perceived stress was 76.1%, 95% CI (72.9–78.8). Older age, educational level, family size, alcohol consumption, smoking, and exposure to traumatic events have shown a statistically significant association with perceived stress.

In this study, 76.1%, 95% CI (72.9–78.8) of participants had perceived stress. A cross-sectional study in West African countries reported a comparable finding that 76.76% of populations had perceived stress [6]. The violence and conflict environments cause massive displacement, financial and family loss, death, and destruction of properties, and hence increase the risk of psychological consequences.

However, this was higher than 65.1% in Nepal [4], 30% in Saudi Arabia [5], 35.9% in Jimma [7], and 61.8% [8] in Southern Ethiopia. The possible factors that explain the variation might be the tool utilized and exposure to multiple traumas. The [5] study used Depression, Anxiety, and Stress Scale DASS-21 tool to measure stress. Besides, in the current study, 79% of participants encountered more than three traumatic events. The increased number of traumas was more likely to raise the magnitude of perceived stress. The ongoing conflict and terrorist acts in the country are also taking a massive toll on the mental and emotional well-being of the community.

Older aged significantly increased the risk of perceived stress. Participants aged above 45 years were more likely to have perceived stress than those aged 18–24 years. Similar to this, a population-based study from Sweden found that stress is very common among elderly persons. The levels of perceived stress increase with increasing age. According to the top tertile of the population, the prevalence of high stress was 7.8% in people aged 81 and older, 7.5% in adults aged 72 to 78, and 6.2% in persons 66 and older [19]. According to estimates in China, elders are also at a significant risk of acquiring post-traumatic stress disorder (PTSD) [20]. This could be because older adults are reluctant to receive advanced warnings or to evacuate during traumatic events, and therefore, may experience greater stress. As cognitive performance degrades with age, higher levels of stress with advancing age may be caused by this decline. Besides, older people are at risk of developing chronic illnesses i.e., diabetes, cardiovascular disease, hearing loss, osteoarthritis, and also other stressors, i.e., a significant loss in capacities and a decline in functional abilities. Further, elders follow negative coping mechanisms, i.e., substance use, that further aggravates the stress.

Perceived stress was significantly higher among participants who didn’t attend formal education. Similarly, a cross-sectional study done by Chekole YA and his colleagues found a statistically significant association between perceived stress and level of qualification [11]. This might be because uneducated participants have poor access to information and hence might have poor knowledge about the damaging nature of war and conflict. In addition, educated people might conduct and explore different types of scientific research regarding its consequence.

There are 52% decreased odds of developing perceived stress among participants who had more than four family sizes. A large family size combined with good family relationships improves their well-being across the adult life course [21]. Diverse family sizes and/or relationships can share love, respect, acceptance, and support for each other. Poor support was a significant predictor of stress among Koshe landslide survivors in Addis Ababa [22]. Family support is a key component of solid relationships and strong psychological health. It involves providing advice, caring, empathy, and material resources through a social network when facing a difficult situation to help individuals cope with stress. Lack of help to compensate for the traumatic experience or to turn to normal daily activities could increase the risk of perceived stress.

It was found that the odds of perceived stress were significantly lower among smokers and alcohol consumers. This might be due to people with traumatic events are using alcohol and cigarette to deal with trauma-related psychological distress, depression, and anxiety. They use to forget war experiences and traumatic life events or emotions. The self-medication hypothesis, substance use as a means to minimize psychiatric symptoms, might also work for alcohol and cigarette users with trauma exposure and perceived stress. In Ethiopia, it is common to use alcohol and cigarette simultaneously; a stimulant that increases energy, concentration, alertness, and sleep disturbances [23]. Consuming alcohol may provide temporary relief from intrusive thoughts but, as the effect wears off, the negative alcohol withdrawal emotions may exacerbate the perceived stress symptoms that someone is attempting to avoid.

Nearly three-fourths (73.4%) of respondents’ personal property was destructed during the war. In addition, 75.9% of participants faced a lack of food and/or water, 37.4% witnessed the murder of a family member/friend, 52.5% were unable to access medical care, and 30% of participants were kidnapped or imprisoned. Furthermore, 15.8% of participants faced one to two traumatic events, whereas 78.8% of them experienced three or more traumas during the war. Stress was two times more common in respondents who encountered more than three traumatic events. This is in line with the study finding in Uganda [24], Nigeria [25], and Southern Ethiopia [26]. Additionally, similar results were found in Palestine, where 88% of participants had gone through personal trauma, 84% had seen trauma inflicted on others, and 88% had watched the demolition of property during the Gaza War [27]. Among Chilean disaster survivors, those with pre-disaster stressors and four or more stresses were more likely to acquire PTSD [28]. In Israel, people who were continually exposed to battle trauma reported having much higher degrees of insecure attachment, perceived stress, and PTSD symptoms. These people had noticeably greater correlations between perceived stress and PTSD symptoms [29]. Exposure to trauma strongly contributes to the development of perceived stress and PTSD. Participants might feel such trauma/loss hard, resulting in increased psychological pain. Following traumatic events, acute stress is a typical response and persistent cases could increase the risk of developing PTSD. The fear and horror engendered by war’s violence disrupts people’s lives, severs relationships and families, and causes emotional distress in both individuals and communities. Trauma exposures may also result in reminders of the event and negative intrusive thoughts like thoughts of revenge and which may contribute to emotional instability or disturbing pattern of thinking and stress. Besides, according to the theory of stress sensitization, rather than fostering resilience, a stressor will render a person more susceptible to the damaging effects of subsequent stressors. As a result, someone who has gone through a lot of stress in their life is more likely to acquire a mental illness [28, 30].

Furthermore, individuals who have experienced trauma also exhibited higher degrees of insecure attachment. The interpretation of subsequent traumatic situations may be influenced by internal working attachment models that are insecure as a result of acute trauma exposure. Continual exposure to traumatic experiences may activate both internal and external stress-relief mechanisms, such as inner security representations, interfering with the regulatory process and escalating stress and distress, according to one theory. A person experiencing trauma may experience extreme shock as well as strong sensations of vulnerability, helplessness, fatigue, and terror. The attachment system may be high level automatically activated under these circumstances [29].

### Limitation

This study was subjected to the following limitations. Due to the cross-sectional nature of the study, it is difficult to determine the direction of causality of the association. We did not consider other psychiatric comorbidities, i.e., anxiety and depression, that could facilitate the development of perceived stress or influence its manifestation and severity. In addition, respondents might not remember whether or not the perceived stress symptoms occurred after the onset of the war and/or conflict. The presence of earlier traumatic experiences might have exacerbated the current disorder related to the war. Some sensitive traumatic events, such as rape or sexual abuse may have been under-reported, especially among females. Social desirability and recall bias might also be present. Moreover, the study was limited to assessing perceived stress in post-war or conflict settings.

## Conclusion and recommendation

This study revealed that more than three-fourths of participants living in post-war settings were found to have perceived stress. Older age, poor level of education, large family size, alcohol consumption, smoking, and the number of traumatic events were found to have a statistically significant association with perceived stress.

Avoiding substance use and psychotherapy that can effectively address the medical, social, and psychological well-being of the population is important to reduce the burden of perceived stress. We suggest perceived stress-focused regular screening by trained health care professionals (Psychiatrists and or Psychologists) and effective community-based treatment approaches of institutions for the current perceived stress and its consequences. Further, more studies, with diverse designs and instruments should be undertaken for more representative findings.

## Supporting information

S1 File English version questionnaire. (DOCX)

S2 File Amharic version questionnaire. (DOCX)

S1 Dataset (SAV)

S1 Checklist STROBE statement—Checklist of items that should be included in reports of observational studies. (DOCX)
