# Supplementary material for: Perceived stress and its associated factors among people living in post-war Districts of Northern Ethiopia: A cross-sectional study
Source: PLoS One. 2022 Dec 28;17(12):e0279571. doi: 10.1371/journal.pone.0279571 (PMC9797080; doi:10.1371/journal.pone.0279571)
Supplement: S1 File — (DOCX) [file pone.0279571.s001.docx]

**English version questionnaire**

Questionnaire code: _____________ Kebele: _____________ House code______________

**Part I: Socio-demographic information**

| **S. No** | **Questions** | **Responses and coding** |
| --- | --- | --- |
| 101 | Age | **________**Years |
| 102 | Sex | 1. Male 2. Female |
| 103 | Residence | 1. Rural 2. Urban |
| 104 | What is your religion? | 1. Christian 2. Muslim |
| 105 | Current marital status | 1. Married/in Union 2. Single 3. Widowed 4. Divorced |
| 106 | Educational status | 1. No formal education 2. Primary education 3. Secondary education 4. College and above |
| 107 | Occupation | 1. Farmer 2. Housewife 3. Gov’t. employee 4. Private employee 5. Daily laborer 6. Other specify__________ |
| 108 | Living arrangement | 1. Live alone 2. Live with my wife or children 3. Live with others |
| 109 | Number of individuals in the household (family size) | ___________ |

**Part II: Pre-existing and behavioral characteristics**

| **S. No** | **Questions** | **Responses and coding** | **Skip** |
| --- | --- | --- | --- |
| 201 | Do you have a pre-existing chronic illness? | 1. Yes 2. No | If No skip to Q203 |
| 202 | If yes, for Q201, which chronic illness do you have?  (You can select more than one option). | 1. Hypertension 2. Diabetes 3. Cardiac disease 4. Kidney disease 5. Respiratory disease 6. HIV/AIDS 7. Others ___________ |  |
| 203 | Have you experienced childhood physical and sexual abuse and neglect? | 1. Yes 2. No |  |
| 204 | Current use of alcohol? | 1. Yes 2. No |  |
| 205 | Current use of khat? | 1. Yes 2. No |  |
| 206 | Current use of tobacco? | 1. Yes 2. No |  |
| 207 | How many hours do you sleep per night? | _________________ |  |

| 208 | Have you ever been diagnosed with mental illness? | 1. Yes 2. No | If No skip to Q210 |
| --- | --- | --- | --- |
| 209 | If yes, for Q208, what is the diagnosis? | 1. Depression 2. Anxiety 3. Substance abuse 4. Perceived stress 5. Other specify___________ |  |
| 210 | Do you know a family member who had ever been diagnosed with mental illness? | 1. Yes 2. No |  |
| 211 | Do you have friends/family who died from mental illness? | 1. Yes 2. No |  |
| 212 | Have you been in a fight with family, friends, or people you love in the past month? | 1. Yes 2. No |  |

**Part III: Trauma-related characteristics**

| **S. No** | **Traumatic events experienced** | **Yes** | **No** |
| --- | --- | --- | --- |
| 301 | Destruction of personal property |  |  |
| 302 | Lack of housing or shelter |  |  |
| 303 | Lack of food or water due to the war |  |  |
| 304 | Witness murder of family member/friends |  |  |
| 315 | Witnessing the murder of a stranger |  |  |
| 306 | Ill health without medical care |  |  |
| 307 | Forced isolation from family/other people |  |  |
| 308 | Tortured or beaten |  |  |
| 309 | Made to accept ideas against the will |  |  |
| 310 | Unnatural death of family, friends, or people you love |  |  |
| 311 | Being abducted or kidnapped or imprisoned |  |  |
| 312 | Rape or sexual abuse |  |  |

**Part IV: Oslo Social Support Scale (OSSS-3)**

| **S. No** | **Questions** | **Response and coding** |
| --- | --- | --- |
| 401 | How many people are so close to you that you can count on them if you have great personal problems? | 1. None 2. 1–2 3. 3–5 4. 5+ |
| 402 | How much interest and concern do people show in what you do? | 1. None 2. Little 3. Uncertain 4. Some 5. A lot |
| 403 | How easy is it to get practical help from neighbors if you should need it? | 1. Very difficult 2. Difficult 3. Possible 4. Easy 5. Very easy |

**Part V: Perceived Stress Scale (PSS)**

| **S. No** | **Your feelings and thoughts in the past month:** | **Never (0)** | **Almost never (1)** | **Sometimes (2)** | **Fairly often (3)** | **Very often (4)** |
| --- | --- | --- | --- | --- | --- | --- |
| 501 | In the last month, how often have you felt that you were unable to control the important things in your life? | 0 | 1 | 2 | 3 | 4 |
| 502 | In the last month, how often have you felt confident about your ability to handle your personal problems? | 4 | 3 | 2 | 1 | 0 |
| 503 | In the last month, how often have you felt that things were going your way? | 4 | 3 | 2 | 1 | 0 |
| 504 | In the last month, how often have you felt difficulties were piling up so high that you could not overcome them? | 0 | 1 | 2 | 3 | 4 |

**Thank you for your co-operation!!!**
