# Supplementary material for: Perceived stress and its associated factors among people living in post-war Districts of Northern Ethiopia: A cross-sectional study
Source: PLoS One. 2022 Dec 28;17(12):e0279571. doi: 10.1371/journal.pone.0279571 (PMC9797080; doi:10.1371/journal.pone.0279571)
Supplement: S2 File — (DOCX) [file pone.0279571.s002.docx]

**የአማሪኛ እትም መጠይቅ**

የመጠይቁ መለያ ቁጥር፡ ______________ ቀበሌ________________ የቤት ኮድ_______________

**ክፍል አንድ፡ የማህበራዊ እና የስነ- ህዝብ መረጃዎች**

| **ተ. ቁ** | **ጥያቄዎች** | **ምላሽና የኮድ መደብ** |
| --- | --- | --- |
| 101 | እድሜዎ ስንት ነው? | **_­­­­­­­­­­________** አመት |
| 102 | ጾታዎ? | 1. ወንድ 2. ሴት |
| 103 | ቋሚ የመኖሪያ አድራሻዎ የት ነው? | 1. ገጠር 2. ከተማ |
| 104 | ሀይማኖትዎ ምንድን ነው | 1. ኦርቶዶክስ 2. ሙስሊም 3. ፕሮቴሰታንት 4. ካቶሊክ 5. ሌላ ይግለጹ ____________ |
| 105 | ወቅታዊ የጋብቻ ሁኔታዎ ምን ይመስላል? | 1. ያገባ(ች) 2. ያላገባ(ች) 3. የትዳር አጋሯ(ሩ)ን በሞት ያጣ(ች) 4. የተፋታ(ች) |
| 106 | የትምህርት ደረጃዎ ምን ያህል ነው? | 1. መደበኛ ትምህርት ያልተማረ(ች) 2. ከ1-8ኛ ክፍል የተማረ(ች) 3. ከ9-12ኛ ክፍል የተማረ(ች) 4. ኮሌጅና ከዛ በላይ የተማረ(ች) |
| 107 | ስራዎ ምንድ ነው? | 1. ግብርና 2. የቤት አመቤት 3. የመንግስት ሰራ 4. የግል ሰራ 5. የቀን ስራ 6. ሌላ ይግለጹ ____________ |
| 108 | ከማን ጋር ነው የሚኖሩት (የአኗኗር ሁኔታዎ)? | 1. ብቻዬን 2. ከባለቤቴ ወይም ከልጆቼ ጋር 3. ከሌሎች ሰዎች ጋር |
| 109 | የቤተሰብዎ አባላት ብዛት (የቤተሰብዎ መጠን) ስንት ነው? | ____________________ |

**ክፍል ሁለት: ቅድመ-ነባራዊ ህመሞች እና የባህሪ ሁኔታዎች**

| **ተ.ቁ** | **ጥያቄዎች** | **ምላሽና የኮድ መደብ** | **ይዝለሉት** |
| --- | --- | --- | --- |
| 201 | ቀደም ሲል በሃኪም የተረጋገጠ ሥር የሰደደ በሽታ አለብዎት? | 1. አዎ 2. የለብኝም | መልስዎ የለብኝም ከሆነ ወደ ተ.ቁ. 203 ይለፉ |
| 202 | ለጥያቄ ቁጥር 201 መልስዎ አዎ ከሆነ፡ ምን አይነት በሽታ አለብዎት?  (ከአንድ በላይ አማራጮችን መምረጥ ይችላሉ) | 1. የደም ግፊት 2. የስኳር በሽታ 3. የልብ በሽታ 4. የኩላሊት በሽታ 5. የመተንፈሻ አካላት በሽታ 6. ኤች አይቪ ኤድስ 7. ሌላ ይገለጹ ____________ |  |
| 203 | በልጅነትዎ አካላዊ ወይንም ወሲባዊ ጥቃት ወይንም መገለል ደርሶቦት ያውቃል? | 1. አዎ 2. አያውቅም |  |
| 204 | አልኮል ነክ መጠጦችን（ጠጅ፡ ጠላ፡ አረቄ፡ ቢራ የመሳሰሉትን）ይጠጣሉ? | 1. አዎ 2. አልጠጣም |  |
| 205 | ጫት ይቅማሉ？ | 1. አዎ 2. አልቅምም |  |
| 206 | ሲጋራ ያጨሳሉ？ | 1. አዎ 2. አላጨስም |  |
| 207 | በአንድ ሌሊት በአማካኝ ለስንት ሰዓት ያህል ይተኛሉ? | ________________ሰአት |  |
| 208 | በሀኪም የተረጋገጠ የአእምሮ ህመም አለብዎት? | 1. አዎ 2. የለብኝም | መልስዎ የለብኝም ከሆነ ወደ ተ.ቁ. 210 ይለፉ |
| 209 | ለጥያቄ ቁጥር 208 መልስዎ አዎ ከሆነ፡ ምን አይነት ህመም አለብዎት? | 1. ድባቴ/ድብርት 2. ጭንቀት 3. ሱስ አስያዥ ንጥረ ነገሮችን መጠቀም 4. የድህረ-አደጋ ጭንቀት (PTSD) 5. ሌላ ይግለጽ _________ |  |
| 210 | በሀኪም የተረጋገጠ የአእምሮ ህመም ያለበት የቤተሰብ አባል አለ? | 1. አዎ 2. የለም |  |
| 211 | በአእምሮ ህመም ምክኒያት የሞቱ ጓደኞች ወይንም የቤተሰብ አባል አለዎት? | 1. አዎ 2. የለም |  |
| 212 | ባለፈው አንድ ወር ውስጥ ከቤተሰብ፣ ከጓደኞችዎ ወይንም ከምትወዷቸው ሰዎች ጋር ተጣልተው ነበር? | 1. 1. አዎ 2. 2. አልተጣላሁም |  |

**ክፍል ሶስት: ከአደጋ ጋር የተያያዙ ክስተቶች**

| **ተ.ቁ** | **ያጋጠሙ የአደጋ ክስተቶች** | **አዎ** | **የለም** |
| --- | --- | --- | --- |
| 301 | የግል ንብረት መውደም |  |  |
| 302 | የመኖሪያ ወይም የመጠለያ እጥረት |  |  |
| 303 | በጦርነቱ ምክንያት የምግብ እና የውሃ እጥረት |  |  |
| 304 | የጓደኛን/ የቤተሰብ አባልን ሲገደል ማየት |  |  |
| 305 | የማያውቁትን ሰው ሲገደል ማየት |  |  |
| 306 | ለህመምዎ/ለበሽታዎ የሕክምና አገልግሎት አለማግኘት |  |  |
| 307 | ከቤተሰብዎ/ከሌሎች ሰዎች በግዳጅ መገለል |  |  |
| 308 | ማሰቃየት፣ መመታት፣መገረፍ |  |  |
| 309 | ያለፍላጎት ሀሳቦችን እንዲቀበሉ መደረግ |  |  |
| 310 | ከተፈጥሮ ውጪ የሆነ የቤተሰብ፣ የጓደኞች ወይም የምትወዳቸው ሰዎች ሞት |  |  |
| 311 | መታሰር፣ ታፍኖ መወሰድ ወይም መታገት |  |  |
| 312 | አስገድዶ መደፈር ወይም ወሲባዊ ጥቃት |  |  |

**ክፍል አራት፡ የኦስሎ ሶስት ማህበራዊ ድጋፍ መለኪያ (Oslo Social Support Scale, OSSS-3)**

| **ተ. ቁ** | **ጥያቄዎች** | **ምላሽና የኮድ መደብ** |
| --- | --- | --- |
| 401 | ትልቅ/ከባድ የግል ችግሮች ቢያጋጥምዎት ምን ያህል ለእርስዎ በጣም ቅርብ የሆኑ ሰዎችን መቁጠር ይችላሉ? | 1. የለም  2. 1–2 ሰዎች  3. 3–5 ሰዎች  4. ከ 5 ሰዎች በላይ |
| 402 | እርስዎ በሚያደርጉት/በሚሰሩት ነገር ሰዎች ምን ያህል ፍላጎት እና አሳቢነት ያሳያሉ? | 1. የለም  2. ትንሽ  3. እርግጠኛ አይደለሁም  4. አንዳንዶች  5. ብዙ ሰዎች |
| 403 | ከጎረቤትዎ ተግባራዊ እርዳታ ለማግኘት ቢፈለጉ ምን ያህል ቀላል ነው? | 1. በጣም አስቸጋሪ  2. አስቸጋሪ  3. ይቻላል  4. ቀላል  5. በጣም ቀላል |

**ክፍል አምስት፡ የተገነዘበ የውጥረት መለኪያ (Perceived Stress Scale)**

| **ተ. ቁ** | **ባለፈው ወር ውስጥ የእርስዎ ስሜቶችና ሀሳቦች፣** | **በፍጹም (0)** | **መቼም (1)** | **አንዳንዴ (2)** | **ብዙ ጊዜ (3)** | **አብዛኛውን ጊዜ (4)** |
| --- | --- | --- | --- | --- | --- | --- |
| 501 | ባለፈው ወር፣ በህይወትዎ ውስጥ አስፈላጊ የሆኑ ነገሮችን መቆጣጠር እንደማይችሉ ምን ያህል ጊዜ ተሰምቶት ነበር? | 0 | 1 | 2 | 3 | 4 |
| 502 | ባለፈው ወር ውስጥ፣ የግል ችግሮችዎን ለመቋቋም ባለዎት አቅም ምን ያህል ጊዜ በራስ መተማመን ተሰምቶት ነበር? | 4 | 3 | 2 | 1 | 0 |
| 503 | ባለፈው ወር ውስጥ፣ ምን ያህል ጊዜ ነገሮች እርስዎ በሚፈልጉት መንገድ እየሄዱ እንደሆን ተሰምቶት ነበር? | 4 | 3 | 2 | 1 | 0 |
| 504 | ባለፈው ወር፣ ምን ያህል ጊዜ ችግሮች በዝተው (ተከምረው) እርስዎ መቋቋም እንደማይችሉ ተሰምቶት ነበር? | 0 | 1 | 2 | 3 | 4 |

**ለትብብርዎ በጣም እናመሰግናለን!!!**
