# Supplementary material for: Perceived stress and its associated factors among people living in post-war Districts of Northern Ethiopia: A cross-sectional study
Source: PLoS One. 2022 Dec 28;17(12):e0279571. doi: 10.1371/journal.pone.0279571 (PMC9797080; doi:10.1371/journal.pone.0279571)
Supplement: S1 Checklist — (DOCX) [file pone.0279571.s004.docx]

STROBE Statement—checklist of items that should be included in reports of observational studies

|  | Item No. | Recommendation | Page  No. | Relevant text from manuscript |
| --- | --- | --- | --- | --- |
| **Title and abstract** | 1 | (*a*) Indicate the study’s design with a commonly used term in the title or the abstract | 1 | Cross-sectional study |
|  |  | (*b*) Provide in the abstract an informative and balanced summary of what was done and what was found | 2 & 3 | The prevalence of perceived stress was 76.1%, 95% CI (72.9-78.8). Age above 45 years (AOR (CI)= 2.45 (1.07-5.62), poor educational level (AOR (CI)= 5.92 (2.36-14.8), large family size (AOR (CI)= 0.48 (0.31-0.74), alcohol consumption (AOR (CI)= 0.63 (0.42-0.94), smoking (AOR (CI)= 0.17 (0.06-0.56), and exposure to multiple traumatic events (AOR (CI)= 2.38 (1.23-4.62) have shown a statistically significant association with perceived stress. |
| Introduction | | | |  |
| Background/rationale | 2 | Explain the scientific background and rationale for the investigation being reported | 2 | There is a conflict between the Ethiopian federal government and Tigray rebels, called the Tigray Peoples Liberation Front, in the Northern part of Ethiopia. War and conflict environments result in long-term physical and psychological consequences. Sexual violence, displacement, malnutrition, death, illness, injury, torture, and disability are some of the physical effects, whereas stress, depression, aggressive behaviours, and anxiety are some of the emotional complications of war. Research is scarce about perceived stress in post-armed conflict regions. Hence, evidence-based interventions are required particularly to monitor mental health disorders. |
| Objectives | 3 | State specific objectives, including any prespecified hypotheses | 2 | To determine the prevalence of perceived stress and its associated factors among people living in post-war situations, Northern Ethiopia, 2022. |
| Methods | | | |  |
| Study design | 4 | Present key elements of study design early in the paper | 6 | Cross-sectional study design |
| Setting | 5 | Describe the setting, locations, and relevant dates, including periods of recruitment, exposure, follow-up, and data collection | 6 | A community-based cross-sectional study was conducted in North Shewa Zone from April 1 to May 15, 2022. |
| Participants | 6 | (*a*) *Cohort study*—Give the eligibility criteria, and the sources and methods of selection of participants. Describe methods of follow-up  *Case-control study*—Give the eligibility criteria, and the sources and methods of case ascertainment and control selection. Give the rationale for the choice of cases and controls  *Cross-sectional study*—Give the eligibility criteria, and the sources and methods of selection of participants | 6 | All people living in war-affected districts of the North Shewa Zone were the source population. All people aged above 18 years irrespective of their sex and who lived for a minimum of six months in the selected districts were included. Individuals who have a hearing problem, severe mental illness or are seriously ill and those who migrated or were not available at the time of invasion/war were excluded from the study. |
|  |  | (*b*) *Cohort study*—For matched studies, give matching criteria and number of exposed and unexposed  *Case-control study*—For matched studies, give matching criteria and the number of controls per case |  |  |
| Variables | 7 | Clearly define all outcomes, exposures, predictors, potential confounders, and effect modifiers. Give diagnostic criteria, if applicable | 7 | Perceived stress was a dependent variable. Independent/exposure variables include socio-demographic factors (age, educational level, marital status, occupation, living arrangement, and family size), pre-existing factors (chronic illness, exposure to childhood trauma, stressful life events, self and family history of mental illness, sleeping hours and substance use), and trauma-related factors (destruction of personal property, lack of food, water and or shelter, witnessing the murder of a family/friends, witnessing the murder of stranger, ill health without medical care, forced isolation from family/other people, tortured or beaten, made to accept ideas against the will, unnatural death of family, friends, or people you love, being abducted or kidnapped or imprisoned, and rape or sexual abuse), and social support. |
| Data sources/ measurement | 8* | For each variable of interest, give sources of data and details of methods of assessment (measurement). Describe comparability of assessment methods if there is more than one group | 7 & 8 | **Perceived stress level:** Measured using the Perceived Stress Scale (PSS). The questions in this scale asked about feelings and thoughts in the last month. PSS was measured with a 5-point Likert- scale ranging from 0=Never, 1=Almost never, 2=Sometimes, 3=Fairly often, 4=Very often) and individuals with higher scores indicated higher perceived stress. A total score of greater than or equal to 8 points was deemed as the cut-off point for categorizing perceived stress associated with war.  **Social support:** Was measured according to the Oslo-3 social support scale which ranges from 3 to 14, those respondents who scored between 3 and 8 were considered to have poor social support, a score of 9–11 was considered as having moderate social support, and a score of 12–14 was considered as having strong social support. |
| Bias | 9 | Describe any efforts to address potential sources of bias | 9 |  |
| Study size | 10 | Explain how the study size was arrived at | 6 | The sample size was determined using Open-Epi version 3.03 statistical software. The following assumptions were made: the power of the study (1-β) to be 80%, 95% confidence interval (CI), 5% margin of error, the prevalence of perceived stress, 51.6% (11), and design effect 2. Then, adding a 10% non-response rate, the sample size equals 845. |

Continued on next page

| Quantitative variables | 11 | Explain how quantitative variables were handled in the analyses. If applicable, describe which groupings were chosen and why | 9 |  |
| --- | --- | --- | --- | --- |
| Statistical methods | 12 | (*a*) Describe all statistical methods, including those used to control for confounding | 9 | The data were cleaned, coded, and entered into Epi-Data version 4.2.2 and exported to SPSS version 25 for analysis. Bivariable and multivariable logistic regression analysis models were run to identify independent determinants of perceived stress. |
|  |  | (*b*) Describe any methods used to examine subgroups and interactions | 9 |  |
|  |  | (*c*) Explain how missing data were addressed | 9 |  |
|  |  | (*d*) *Cohort study*—If applicable, explain how loss to follow-up was addressed  *Case-control study*—If applicable, explain how matching of cases and controls was addressed  *Cross-sectional study*—If applicable, describe analytical methods taking account of sampling strategy | 9 |  |
|  |  | (*e*) Describe any sensitivity analyses |  |  |
| Results | | | | |
| Participants | 13* | (a) Report numbers of individuals at each stage of study—eg numbers potentially eligible, examined for eligibility, confirmed eligible, included in the study, completing follow-up, and analysed | 10 | A total of 812 participants were interviewed, making a 96.1% response rate. |
|  |  | (b) Give reasons for non-participation at each stage |  |  |
|  |  | (c) Consider use of a flow diagram |  |  |
| Descriptive data | 14* | (a) Give characteristics of study participants (eg demographic, clinical, social) and information on exposures and potential confounders | 10 | The mean (±SD) age of respondents was 31.13 ± 9.62 and ranged from 18 to 75 years. More than half, 428 (52.7%) of the respondents were found in the age range of 25 – 34 years (Table 1). |
|  |  | (b) Indicate number of participants with missing data for each variable of interest | 10 |  |
|  |  | (c) *Cohort study*—Summarise follow-up time (eg, average and total amount) |  |  |
| Outcome data | 15* | *Cohort study*—Report numbers of outcome events or summary measures over time |  |  |
|  |  | *Case-control study—*Report numbers in each exposure category, or summary measures of exposure |  |  |
|  |  | *Cross-sectional study—*Report numbers of outcome events or summary measures | 10 – 15 |  |
| Main results | 16 | (*a*) Give unadjusted estimates and, if applicable, confounder-adjusted estimates and their precision (eg, 95% confidence interval). Make clear which confounders were adjusted for and why they were included | 16 & 17 | Respondents aged 45 years and above were two times more likely to develop perceived stress compared to those aged between 18 – 24 years (AOR (CI)= 2.45 (1.07-5.62). There were six times more odds of perceived stress among uneducated participants (AOR (CI)= 5.92 (2.36-14.8). Perceived stress was also two times more common in respondents who encountered more than three traumatic events (AOR (CI)= 2.38 (1.23-4.62). The odds of perceived stress were 37% lower among alcohol consumers than their counterparts (AOR (CI)= 0.63 (0.42-0.94). Additionally, smoking decreased the risk of perceived stress by 83% (AOR (CI)= 0.17 (0.06-0.56). Further, there are 52% decreased odds of developing perceived stress among participants who had more than four family sizes (AOR (CI)= 0.48 (0.31-0.74) (Table 4). |
|  |  | (*b*) Report category boundaries when continuous variables were categorized | 10 – 17 |  |
|  |  | (*c*) If relevant, consider translating estimates of relative risk into absolute risk for a meaningful time period |  |  |

Continued on next page

| Other analyses | 17 | Report other analyses done—eg analyses of subgroups and interactions, and sensitivity analyses |  |  |
| --- | --- | --- | --- | --- |
| Discussion | | | | |
| Key results | 18 | Summarise key results with reference to study objectives | 24 & 25 | The prevalence of perceived stress was 76.1%, 95% CI (72.9-78.8). Older age, educational level, family size, alcohol consumption, smoking, and exposure to traumatic events have shown a statistically significant association with perceived stress. |
| Limitations | 19 | Discuss limitations of the study, taking into account sources of potential bias or imprecision. Discuss both direction and magnitude of any potential bias | 22 | This study was subjected to the following limitations. Due to the cross-sectional nature of the study, it is difficult to determine the direction of causality of the association. We did not consider other psychiatric comorbidities, i.e., anxiety and depression, that could facilitate the development of perceived stress or influence its manifestation and severity. In addition, respondents might not remember whether or not the perceived stress symptoms occurred after the onset of the war and/or conflict. The presence of earlier traumatic experiences might have exacerbated the current disorder related to the war. Some sensitive traumatic events, such as rape or sexual abuse may have been under-reported, especially among females. Social desirability and recall bias might also be present. Moreover, the study was limited to assessing perceived stress in post-war or conflict settings. |
| Interpretation | 20 | Give a cautious overall interpretation of results considering objectives, limitations, multiplicity of analyses, results from similar studies, and other relevant evidence | 19 – 21 |  |
| Generalisability | 21 | Discuss the generalisability (external validity) of the study results | 22 |  |
| Other information | |  | | |
| Funding | 22 | Give the source of funding and the role of the funders for the present study and, if applicable, for the original study on which the present article is based | 23 & 24 | This study was funded by Debre Berhan University, Asrat Woldeyes Health Science Campus. Every phase of the study was evaluated and monitored by the Asrat Woldeyes Health Science Campus. The funder has no role in the design, data collection, analysis, preparation of the manuscript, and decision to publish. |

*Give information separately for cases and controls in case-control studies and, if applicable, for exposed and unexposed groups in cohort and cross-sectional studies.

**Note:** An Explanation and Elaboration article discusses each checklist item and gives methodological background and published examples of transparent reporting. The STROBE checklist is best used in conjunction with this article (freely available on the Web sites of PLoS Medicine at http://www.plosmedicine.org/, Annals of Internal Medicine at http://www.annals.org/, and Epidemiology at http://www.epidem.com/). Information on the STROBE Initiative is available at www.strobe-statement.org.
